# Effects of Modified Sanzi Yangqin Decoction on Tyrosine Phosphorylation of IRS-1 in Skeletal Muscle of Type 2 Diabetic Rats

**DOI:** 10.1155/2018/7092140

**Published:** 2018-03-12

**Authors:** Ya Wang, Xiaojin La, Chunyu Tian, Yushan Dong, Feng Qi, Changlong Qiu, Xiujuan Gao, Ji'an Li

**Affiliations:** ^1^Graduate School, Tianjin University of Traditional Chinese Medicine, Tianjin 300193, China; ^2^College of Traditional Chinese Medicine, North China University of Science and Technology, Tangshan 063210, China

## Abstract

This study aimed to investigate the effect of Modified Sanzi Yangqin Decoction on tyrosine phosphorylation of insulin receptor substrate 1 (IRS-1) in skeletal muscle of type 2 diabetic rats. The rat model of type 2 diabetes was induced by high-fat diet and multiple low-dose streptozotocin injections. Diabetic model rats were randomly divided into 5 groups: the model control group, the metformin group, and Modified Sanzi Yangqin Decoction groups of low, medium, and high doses. OGTT was conducted every two weeks during treatment period. At the end of the treatment, the fasting blood glucose (FBG) level and the fasting C-peptide level were measured to calculate insulin resistance index. The levels of IRS-1, p-IRS-1^(Tyr895)^, and protein tyrosine phosphates 1B (PTP1B) in skeletal muscle were also measured. Modified Sanzi Yangqin Decoction significantly reduced the FBG level, increased the fasting C-peptide level, and lowered the insulin resistance index in type 2 diabetic rats. It also significantly increased the protein level of p-IRS-1^(Tyr895)^ and reduced the PTP1B protein level in skeletal muscle of type 2 diabetic rats. Modified Sanzi Yangqin Decoction increases tyrosine phosphorylation of IRS-1 in skeletal muscle of type 2 diabetic rats, which results from the increase of p-IRS-1^(Tyr895)^ protein and is related to the suppression of PTP1B protein.

## 1. Introduction

Type 2 diabetes mellitus (T2DM) is one of the world's most common health issues and its incidence has been growing at a particularly high rate [1, 2]. T2DM and its complications have high risk of disabilities and mortality rates [1]. About 425 million people worldwide are estimated to have diabetes and some 629 million people are projected to develop diabetes by 2045 [1]. Insulin resistance (IR) is an important pathological condition of T2DM, in which the body is still able to produce insulin but the cells become resistant to its effect, making insulin ineffective in regulating blood sugar levels. Over time, insulin levels may become insufficient as well. Both the insulin resistance and deficiency lead to high blood glucose levels. Therefore, increasing insulin sensitivity is an important and effective therapy for T2DM [3].

The underlying cause of insulin resistance is multifaceted. Obesity is commonly associated with insulin resistance. Specifically, the level of free fatty acids, also referred to as nonesterified acids (NEFA), is increased in obesity and plays an important role for the association between obesity and type 2 diabetes [4]. Chronic insulin resistance is also related to diet-induced inflammation, which interrupts insulin's normal function by disrupting the signaling mechanism [5]. As a promising alternative therapy for treating diabetes, the traditional Chinese medicine theory attributes the fundamental pathogenesis mechanism of T2DM to kidney deficiency and phlegm turbidity. Middle-aged and senior adults are at the highest risk for type 2 diabetes [6], and they are also at the age stage of kidney-qi deficiency [7]. Additionally, most patients diagnosed with T2DM are considered obese [8] and tend to have high-sugar/high-fat diet, which is usually associated with phlegm turbidity [9]. Interestingly, the syndrome of phlegm turbidity in TCM usually causes chronic inflammation, which, based on modern scientific research, contributes to the pathogenesis of insulin resistance [9, 10]. The kidney deficiency and phlegm turbidity pathogenesis theory were also supported by the clinical studies by Pan et al., which showed that most common syndromes of T2DM are kidney deficiency, phlegm dampness, and blood stasis [11, 12].

Based on the above pathogenesis mechanism, one possible TCM therapy of diabetes is to supplement kidney and remove phlegm turbidity. We modified Sanzi Yangqin Decoction, a classic formula recorded in* Han's Medicine Theory,* with the addition of dodder and glossy privet. Sanzi Yangqin Decoction consists of perilla fruit, radish seed, and white mustard seed. Perilla fruit is rich in alpha-linolenic acid which exerts an endothelial protective effect against high glucose injury via PI3K/Akt pathway [13]. Previous pharmacological experiments showed that oleanolic acid, an important ingredient in glossy privet fruit, has hypoglycemic effects [14, 15]. In clinical practices, Modified Sanzi Yangqin Decoction worked quite well for treating diabetic patients (Supplementary Table 1).

What remains unclear is the molecular mechanism of the effectiveness of Modified Sanzi Yangqin Decoction. The insulin signal transduction pathway begins with the bond of insulin to insulin receptors on the surface of skeletal muscle cells. The tyrosine kinase activity of the insulin receptors is stimulated, leading to the tyrosine phosphorylation of IRS-1 [16]. Tyrosine-phosphorylated IRS-1 further triggers the activation of a series of downstream molecules to transport blood glucose into cells for synthesis of muscle glycogen or oxidation decomposition [16]. Among many components involved in the insulin transduction pathway, PTP1B is a key negative regulation factor which blocks the tyrosine phosphorylation of IRS-1. It was shown that overexpression of PTP1B increases the dephosphorylation of IRS-1 and IRS-2 and downregulates postreceptor signal transduction [17]. On the contrary, inhibiting the overexpression of PTP1B can increase phosphorylation levels of insulin receptor and IRSs at tyrosine residues, strengthen insulin signal transduction, and improve IR [18, 19].

In this study, we developed rat model of type 2 diabetes mellitus and treated them with varying dosage of Modified Sanzi Yangqin Decoction and metformin. We evaluated the improvement of insulin resistance and studied the effect of Modified Sanzi Yangqin Decoction on IRS-1, p-IRS-1^(Tyr895)^, and PTP1B, key molecules involved in the insulin signal transduction pathway.

## 2. Materials and Methods

### 2.1. Animals

SPF Wistar rats [male, 200 ± 10 g] were provided by Tianjin Shanchuanhong Experimental Animal Science and Technology Ltd. [permit number: SCXK(Jin) 2009-0001; certificate number: 0253756]. The rats were kept in Laboratory Animal Center of North China University of Science and Technology (MY10DXK07, Tangshan, China). Rats were kept in standard living conditions [room temperature (21 ± 2)°C, regular humidity (50 ± 10%), and 12 h dark-light cycle]. All experiments were performed in compliance with the guidelines for the care and use of laboratory animals as approved by the Animal Ethics Committee of North China University of Science and Technology.

### 2.2. Drugs

Modified Sanzi Yangqin Decoction was prepared from perilla fruit* (Perilla frutescens)*, radish seed* (Semen Raphani)*, white mustard seed* (Semen Sinapis)*, dadder seed (*Cuscuta chinensis* Lam.), and ligustrum seed* (Fructus Ligustri Lucidi)* from Beijing Tong Ren Tang Chinese Medicine Co. Ltd. in Tangshan. After identification confirmed by Chunyu Tian (Institute of Traditional Chinese Medicine, North China University of Science and Technology), these herbs were mixed in the proportion of 1 : 1 : 1 : 1 : 1 and extracted by boiling water twice (2 h each time). The decoction was concentrated and dehydrated in vacuo (70°C) and ground into powder. The extraction rate of the dry extract was 20.29% (actual dry powder/actual crude herbs). The prepared powder was kept at 0°C–4°C and dissolved with purified water into suspension of different concentrations. Metformin hydrochloride tablets (500 mg/tablet) were obtained from Shi Bao Sino-US Ltd. in Shanghai (#H20023370).

### 2.3. Reagents

The reagents used in this study include the following: streptozotocin (STZ), Sigma Inc., St Louis, United States (#B56981); rat C-peptide ELISA kit, Shanghai Jianglai Bio-Technology Co., Ltd., Jiangshu, China (#96T/48T); nitrocellulose (NC) membrane, the Merck Millipore, Massachusetts, United States (#HATF00010); primary antibody of PTP1B, Abcam, Cambridge, United Kingdom (#ab2009); the following products were from Cell Signaling Technology, Inc., Massachusetts, United States: primary antibody of p-IRS-1^(Tyr895)^ (#3070) and primary antibody of IRS-1 (#3407). The following products were from Bi Yuntian biological company, Shanghai, China: western and IP cell lysis buffer (#P0013), PMSF (100 mM) (#ST506), BCA kit (enhanced) (#P0010), SDS-PAGE Gel Preparation Kit (#P0012A), Prestained Protein Molecular Weight Marker (#P0068), primary antibody of GAPDH (#AG019), primary antibody of *β*-actin (#AF0003), Horseradish Peroxidase- (HRP-) Conjuncted Goat Anti-Mouse IgG (H+L) (#A0216), and HRP-Conjuncted Goat Anti-Rabbit IgG (H+L) (#A0208).

### 2.4. Main Instruments

The main instruments were as follows: IKA-T10 basic homogenizer (IKA, Staufen, Germany); low-temperature high-speed centrifuge R134A (Eppendorf, Hamburg, Germany); Infinite F50 microplate reader (Tecan Group Ltd., Mannedorf, Switzerland); multifunction electrophoresis apparatus (Liuyi Instrument Factory, Beijing, China); Ice Maker 2BE-70-25 (ZIGERA, Germany); horizontal shaking table (GFL, United States); cassette and X film (Kodak, Japan).

### 2.5. Induction of T2DM Rat Model

Eighty male Wistar rats were fed with standard diet for one week to adapt to the environment. 10 rats were randomly selected as normal control group (Norm) and fed with standard diet. The remaining rats were fed with high-fat diet (http://shop.ys-bio.com/goods-495.html, Research Diets D12492 60 kcal% fat containing 60% fat, 20% protein, and 20% carbohydrates from Beijing Hua Fu Kang Bioscience Co., Ltd.) for 6 weeks. The rats fed with high-fat diet were intraperitoneally injected with streptozotocin (STZ) solution twice (40 mg·kg^−1^ and 30 mg·kg^−1^) [20]. The rats in Norm were injected with the same dose of buffer solution. All rats were fasted for 12 hours before each injection of STZ. On day 7 after the final injection of STZ, all rats were tested for the levels of fasting blood glucose (FBG) and 2 h postprandial blood glucose (2 hPG). 59 rats were considered diabetic with FBG ≥ 11.1 mmol·L^−1^ or 2 hPG ≥ 16.7 mmol·L^−1^ [21].

### 2.6. Grouping and Drug Administration

With the exclusion of 9 rats with 2 hPG > 30.0 mmol·L^−1^, the remaining 50 diabetic rats were randomly divided into 5 groups: the model control group (T2DCn), the metformin group (MET), and Modified Sanzi Yangqin Decoction groups of low, medium, and high dose (MSYDl, MSYDm, and MSYDh, resp.), with 10 rats in each group. According to the drug dosage conversion formula between rats and human [22], rats in Norm and T2DCn were given purified water and rats in MET were given metformin hydrochloride suspension 0.1 g·(kg·d)^−1^. The rats in the groups of MSYDl, MSYDm, and MSYDh were treated with powder suspensions of Modified Sanzi Yangqin Decoction equal to the crude drugs of 3.0, 6.0, and 12.0 g·(kg·d)^−1^, respectively. Rats were weighed once a week for adjusting drug dosage in the 6 weeks' intervention period.

### 2.7. Sampling and Main Outcome Measures

At the end of the six-week treatment period, rats were anesthetized with chloral hydrate (10%, ip) after 12 h fasting, and blood samples were collected from the abdominal aorta. Rectus femoris muscle in the left limb was quickly removed, cleaned with saline rinse, and frozen immediately in liquid nitrogen for cryopreservation.

### 2.8. OGTT and AUC Calculation

In the sixth week after treatment (on day 40), the rats of normal and diabetic groups were orally treated with 2 g/kg of glucose. The blood glucose levels in blood samples collected from tail veins were measured at 0, 30, 60, and 120 minutes after glucose loading. The AUC was calculated by the following formula: AUC (mmol/L × min)* * = * *1/2 × (BG 0 min + BG 30 min) × 30 min + 1/2 × (BG 30 min + BG 60 min) × 30 min + 1/2 × (BG 60 min + BG 120 min) × 60 min.

### 2.9. FBG, Fasting C-Peptide, and Insulin Resistance Index

The blood samples from the abdominal aorta were centrifuged to collect serum. One drop of arterial blood was sampled through an arterial catheter for FBG measurement. FBG level was measured by glucose-oxygenation-enzyme-method with glucose meter. Fasting C-peptide was measured by ELISA with Tecan microplate reader.

The values of FBG and fasting C-peptide were used to calculate insulin resistance index according to the formula below [23]:

(1) $$\begin{array}{c} \text{Homa-IR(CP)}=1.5+\text{FBG}\times \frac{\text{fasting CP}}{2800}, \end{array}$$

where FBG is fasting blood glucose (mmol/L) and CP is C-peptide (pmol/L)

### 2.10. Western Blot for Protein Contents of p-IRS-1^(Tyr895)^, IRS-1, and PTP1B in Skeletal Muscle

The skeletal muscle samples were mashed with homogenizer and cells were lysed in lysis buffer. After centrifugation, the supernatant was collected, and the concentration of each group was measured with bicinchoninic acid (BCA) protein assay kit. After mixing with SDS-sample buffer, cell lysates were separated by SDS-PAGE and transferred onto NC membrane and were incubated with antibody against p-IRS-1^(Tyr895)^, IRS-1, PTP1B, GAPDH, or *β*-actin. After incubation with appropriate peroxidase-conjugated secondary antibodies, the immunoreactive bands were visualized by enhanced chemiluminescence (ECL) reagents and imaged using X-ray film. The imaged bands were quantified with Pro Plus 6.0 medical image analysis software (Cybernetics Media, USA).

### 2.11. Statistical Analysis

All data were expressed as $\overset{-}{x}\pm \text{SD}$ and analyzed with SPSS13.0 software. Differences of parametric data among several groups were determined by one-way analysis of variance (ANOVA). Any differences with *p* < 0.05 were considered significant (two-tailed).

## 3. Results

### 3.1. Effects of Modified Sanzi Yangqin Decoction on General Conditions

**Figure 1:**
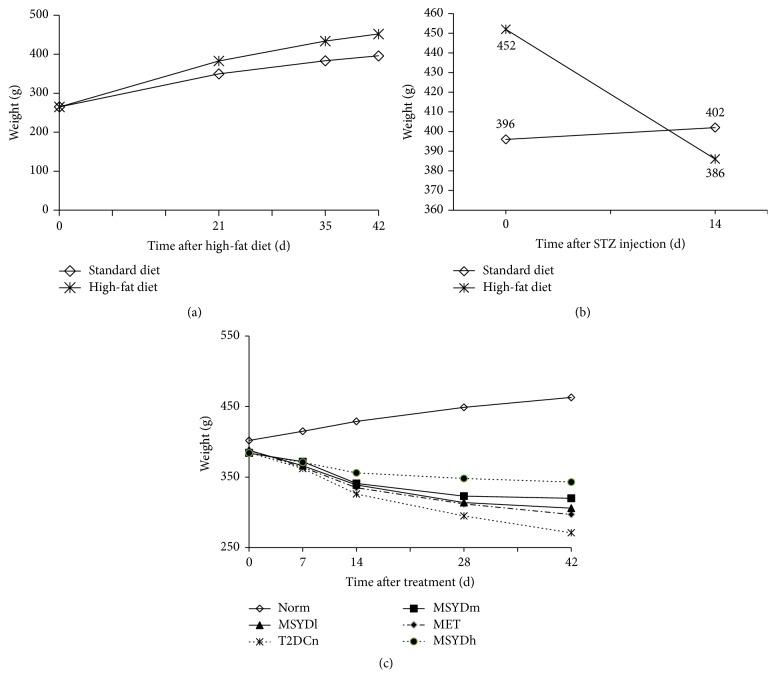
Body weight of rats in different stages. (a) The body weight of rats increased steadily. The high-fat fed rats gained weight more rapidly than those fed with standard diet. (b) The body weight of rats fed with high-fat diet was lost significantly after STZ injection. (c) The body weight of rats in Norm increased steadily, and that of diabetic rats decreased progressively. With treatment, the weight loss of diabetic rats slowed down, especially in MSYDh. STZ, streptozotocin; Norm, normal control group; T2DCn, model control group; MET, metformin group; MSYDl, low-dose Modified Sanzi Yangqin Decoction group; MSYDm, medium-dose Modified Sanzi Yangqin Decoction group; MSYDh, high-dose Modified Sanzi Yangqin Decoction group.

Rats in Norm (normal control group) had the following characteristics: normal daily food and water intake, normal excretion of urine and stool, smooth fur, in good mental state, and swift action; rats in T2DCn (model control group) the following characteristics: polydipsia, polyuria, loose stools, rough and dull fur, mental fatigue, slow in reacting, arched back, and weight loss; compared with those in T2DCn, rats in Modified Sanzi Yangqin Decoction groups showed significantly relieved symptoms of polydipsia, polyuria, and weight loss (Figure 1), with glossy fur and swift response. The rats in MET also showed improvement of symptoms and signs, although not as significant as those in the Modified Sanzi Yangqin Decoction groups.

### 3.2. Effects of Modified Sanzi Yangqin Decoction on FBG and 2 hPG Levels

**Figure 2:**
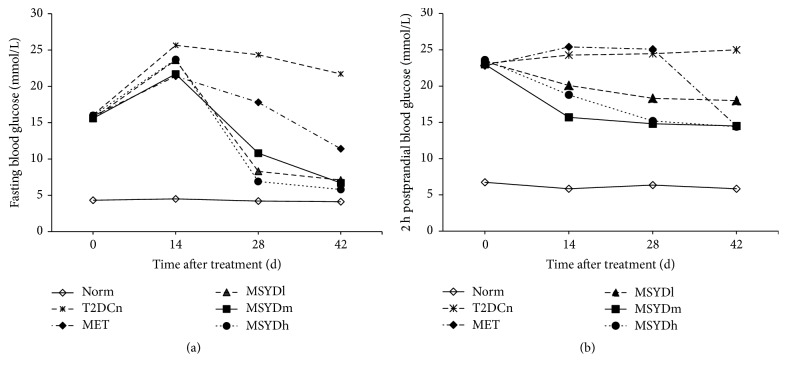
Levels of fasting blood glucose and 2 h postprandial blood glucose. (a) The levels of fasting blood glucose. (b) The levels of 2 h postprandial blood glucose. Average levels of 10 subjects in each group are shown. Statistical significance was calculated with one-way ANOVA. Norm, normal control group; T2DCn, model control group; MET, metformin group; MSYDl, low-dose Modified Sanzi Yangqin Decoction group; MSYDm, medium-dose Modified Sanzi Yangqin Decoction group; MSYDh, high-dose Modified Sanzi Yangqin Decoction group.

As shown in Figure 2, before drug administration, the FBG and 2 hPG levels of the diabetic rats were significantly higher than those of the rats in Norm (*p* < 0.05), but there was no statistically significant difference between groups of diabetic rats (*p* > 0.05). On day 14 after drug treatment, FBG levels were significantly increased in all diabetic rats (*p* < 0.05); 2 hPG levels were significantly reduced in MSYD groups (*p* < 0.05), but it remained at high levels in MET and T2DCn. On day 28 after treatment, the FBG levels in treatment groups were significantly decreased compared to those of T2DCn (*p* < 0.05); the decline of 2 hPG level continued in each of the MSYD groups (*p* < 0.05), but there was no significant change in 2 hPG level in MET. On day 42, the levels of FBG and 2 hPG were significantly reduced in all treatment groups (*p* < 0.05). The FBG level in T2DCn decreased gradually after day 14, but was still quite high (over 20 mmol·L^−1^).

### 3.3. Effects of Modified Sanzi Yangqin Decoction on Glucose Tolerance

**Figure 3:**
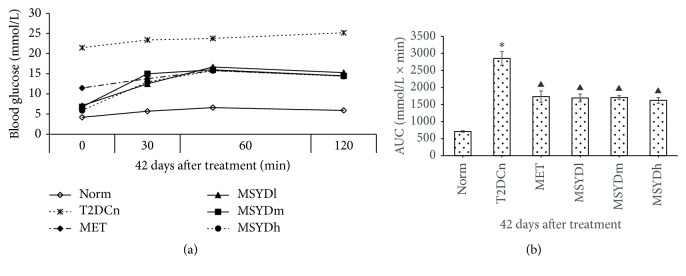
Effects of Modified Sanzi Yangqin Decoction on AUC of OGTT in diabetic rats. (a) The changes in blood glucose concentration during OGTT in diabetic rats. (b) The areas under the curve of the glucose response in type 2 diabetic rats. Average levels of 10 subjects in each group are shown. Statistical significance was calculated with one-way ANOVA. Norm, normal control group; T2DCn, model control group; MET, metformin group; MSYDl, low-dose Modified Sanzi Yangqin Decoction group; MSYDm, medium-dose Modified Sanzi Yangqin Decoction group; MSYDh, high-dose Modified Sanzi Yangqin Decoction group. Compared with Norm, ^*∗*^*p* < 0.05; compared with T2DCn, ^▲^*p* < 0.05.

As shown in Figure 3(a), after 42 days of treatment, the diabetic rats treated with MSYD have blood glucose levels close to normal rats in Norm. The diabetic rats treated with metformin have blood glucose levels between the MSDY treated rats and the diabetic rats without any treatment. After oral glucose loading, the blood glucose level in untreated diabetic rats (T2DCn) increased steadily. For diabetic rats treated with metformin (MET) and MSYD (MSYDl, MSYDm, and MSYDh), after glucose loading, their blood glucose level increased initially, reached peak level after 60 minutes, and dropped slightly by 120 minutes. These were no significant differences between metformin treatment and difference dosages of Modified Sanzi Yangqin Decoction. The AUC values of diabetic rats treated with metformin and MSYD are significantly smaller than those of diabetic rats without any treatment (*p* < 0.05, Figure 3(b)).

### 3.4. Effects of Modified Sanzi Yangqin Decoction on Fasting C-Peptide and IR Index

Table 1 shows the effect of MSYD on the level of fasting blood glucose, fasting C-peptide, and the extent of IR in type 2 diabetic rats. Compared with Norm, a lower level of fasting C-peptide content was observed in diabetic rats of T2DCn (*p* < 0.05). Increased levels of C-peptide were observed in each treatment group on day 42 after treatment, especially in MSYDh (*p* < 0.05). Statistical analysis also revealed that MSYD mitigated insulin resistance in diabetic rats and its anti-IR effect was remarkably better than metformin (*p* < 0.05). But there was no significant difference between each MSYD group (*p* > 0.05).

### 3.5. Effects of Modified Sanzi Yangqin Decoction on Protein Levels of p-IRS-1, IRS-1, and PTP1B in Skeletal Muscle of T2DM Rats

**Figure 4:**
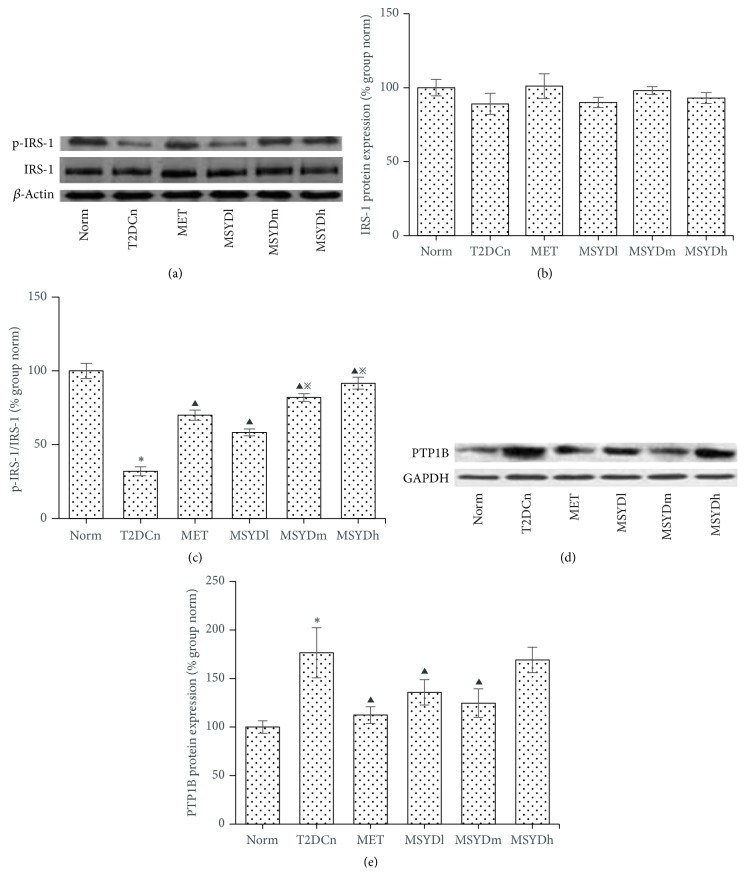
Protein expression of IRS-1, p-IRS-1, and PTP1B in rat skeletal muscle. Results from a representative experiment in triplicate. Data are presented as means ± SEMs. Statistical significance was calculated based on one-way ANOVA. Norm, normal control group; T2DCn, model control group; MET, metformin group; MSYDl, low-dose Modified Sanzi Yangqin Decoction group; MSYDm, medium-dose Modified Sanzi Yangqin Decoction group; MSYDh, high-dose Modified Sanzi Yangqin Decoction group. Compared with Norm, ^*∗*^*p* < 0.05; compared with T2DCn, ^▲^*p* < 0.05; compared with MET, ^*※*^*p* < 0.05.

The total IRS-1 protein levels in the muscle samples are similar between rats from different groups (*p* > 0.05) (Figures 4(a) and 4(b)); however the phosphorylated form of IRS-1 in untreated diabetic rats is significantly lower than that in the normal control group and the treatment groups (*p* < 0.05), with MSYDh having the highest level of p-IRS-1 (Figures 4(a) and 4(b)). The PTP1B, a negative regulator of insulin signaling, shows significantly lower level in the MSYD and metformin group than that in the untreated diabetic group (T2DCn) (*p* < 0.05), with the exception of MSYDh (Figures 4(d) and 4(e)).

## 4. Discussion

Insulin resistance (IR) is a major defect in type 2 diabetes mellitus (T2DM), referring to the reduced sensitivity of the target tissues in response to insulin [24]. Skeletal muscle is the main tissue for glucose metabolism, as 80% of insulin-mediated glucose uptake and utilization occur in skeletal muscle [25]. Insulin resistance in skeletal muscle IR is the initiating and primary defect in T2DM [26, 27].

In this study, high-fat diet combined with multiple low-dose STZ injections was used to develop stable animal models of type 2 diabetes mellitus [20]. The rats fed with high-fat diet develop insulin resistance [28]. Low-dose STZ induces a mild impairment of insulin secretion similar to the characteristics of later-stage type 2 diabetes [29]. The rat model showed abnormally high levels of FBG, 2 hPG, and insulin resistance index, and impaired glucose tolerance was diagnosed, indicating that the rat model of T2DM was induced successfully.

We observed that p-IRS-1^(Tyr895)^ protein expression was decreased and PTP1B protein expression was increased in the skeletal muscle of T2DM model rats, suggesting the existence of defects in insulin signal transduction. Through further evaluation, it was observed that the tyrosine phosphorylation level was significantly decreased in skeletal muscle of T2DM model rats, which was related to the reduction of p-IRS-1^(Tyr895)^ protein and the elevation of PTP1B protein expression. Therefore, it is important to increase phosphorylation level of IRS-1 at tyrosine residues and inhibit the overexpression of PTP1B for IR improvement in skeletal muscle.

After treatment with Modified Sanzi Yangqin Decoction for six weeks, the levels of FBG and 2 hPG were reduced, and insulin resistance index was lowered in T2DM model rats. There were two observations worth pointing out. First, the FBG level was increased in all diabetic rats on day 14 after drug treatment. Previous studies reported similar pattern of blood glucose variation in STZ-induced diabetic rats [20]. The STZ-induced loss of *β*-cell mass is progressive [30]; therefore FBG continues to rise throughout the first few weeks, and the hyperglycemia becomes stable gradually. Interestingly, the increase of FBG level was slower in drug treatment groups than untreated diabetic rate group, indicating Modified Sanzi Yangqin Decoction or metformin can slow the increase of blood glucose, although they cannot stop the increasing trend in the initial stage, possibly due to the residual effect of STZ. The other observation was that the hypoglycemic effect of metformin was not as obvious as expected compared with Modified Sanzi Yangqin Decoction. We hypothesized that it was possibly due to the plasma concentration of metformin which may be very low at the time of OGTT since the metformin suspension was administrated to diabetic rats after OGTT. These observations confirmed the effectiveness of Modified Sanzi Yangqin Decoction in alleviating insulin resistance and treating diabetes.

In regard of the effect of Modified Sanzi Yangqin Decoction on the insulin signaling pathway, we observed that the level of phosphorylated form of IRS-1 (p-IRS-1^(Tyr895)^) was increased with treatment while the overall level of IRS-1 remained similar across different experimental groups. The increase in p-IRS-1 level was accompanied by the decrease of PTP1B level in skeletal muscle. These observations suggested PTP1B as one potential target of Modified Sanzi Yangqin Decoction, which likely functions by suppressing PTP1B expression and therefore restoring normal phosphorylation of IRS-1 and the insulin signal transduction. There are many other molecules involved in the signal transduction of insulin, such as the downstream PI3K/AKT pathway. To further understand the molecular mechanism of Modified Sanzi Yangqin Decoction, it will be interesting to study if it has any effect on other molecules involved in insulin signaling pathway.

## 5. Conclusions

Modified Sanzi Yangqin Decoction reduces the levels of fast blood glucose and 2 h postprandial blood glucose in T2DM model rats. Modified Sanzi Yangqin Decoction relieves the insulin resistance by increasing IRS-1 phosphorylation at tyrosine in skeleton muscle of T2DM rats, which results from the increased level of p-IRS-1^(Tyr895)^ protein and is related to the suppression of the PTP1B protein.

## Figures and Tables

**Table 1:** Fasting C-peptide, insulin, and fasting blood glucose in type 2 diabetic rats.

	FBG (mmol**·**L^−1^)	C-P (pg**·**mL^−1^)	Homa-IR (CP)
Norm	4.5 ± 0.39	4.20 ± 0.130	0.0072 ± 0.00059
T2DCn	22.8 ± 2.21^*∗*^	3.77 ± 0.103^*∗*^	0.0312 ± 0.00287^*∗*^
MET	11.5 ± 1.57^▲^	4.31 ± 0.327^▲^	0.0182 ± 0.00297^▲^
MSYDl	7.5 ± 1.05^▲*※*^	4.35 ± 0.240^▲#^	0.0122 ± 0.00287^▲*※*^
MSYDm	7.2 ± 0.89^▲*※*^	4.25 ± 0.397^▲#^	0.0115 ± 0.00149^▲*※*^
MSYDh	6.3 ± 0.94^▲*※*^	4.59 ± 0.310^▲#^	0.0108 ± 0.00198^▲*※*^
*F*	104.407	142.196	92.972
*p*	0.000	0.000	0.000

Data were expressed as mean ± SD, *n*  = 10; compared with Norm, ^*∗*^*p* < 0.05; compared with T2DCn, ^▲^*p* < 0.05; compared with MET, ^*※*^*p* < 0.05, ^#^*p* > 0.05.

## Supplementary Materials

Supplementary Materials Supplementary Table 1: FPG and 2 hPG in Modified Sanzi Yangqin Decoction group and metformin group.
