# Supplementary material for: Effects of Modified Sanzi Yangqin Decoction on Tyrosine Phosphorylation of IRS-1 in Skeletal Muscle of Type 2 Diabetic Rats
Source: Evid Based Complement Alternat Med. 2018 Mar 12;2018:7092140. doi: 10.1155/2018/7092140 (PMC5867590; doi:10.1155/2018/7092140)
Supplement: Supplementary Materials — Supplementary Table 1: FPG and 2 hPG in Modified Sanzi Yangqin Decoction group and metformin group. [file 7092140.f1.doc]

Supplementary Table 1 FPG and 2hPG in Modified Sanzi Yangqin Decoction group and metformin group

| Indicators | Modified Sanzi Yangqin Decoction group (means ± SD) | metformin group  (means ± SD) | P |
| --- | --- | --- | --- |
| FPG (mmol/L) | 7.52±1.36 | 7.45±1.40 | 0.736 |
| 2hP G (mmol/L) | 11.43±1.65 | 11.53±2.02 | 0.714 |

During the period between March 2016 to March 2017, an observational study involving 120 diabetic cases were conducted. Among the 120 patients, 60 were treated with Modified Sanzi Yangqin Decoction, and the other 60 were treated with metformin. The levels of FPG and 2hPG were measured after 4 weeks’ treatment. Modified Sanzi Yangqin Decoction shows therapeutic effects comparable to that of metformin. The study was conducted in Metabolic Department at Qian’an Hospital of Traditional Chinese Medicine, Department of Endocrinology at the First Teaching Hospital of Tianjin University of Traditional Chinese Medicine and Metabolic Department at Tangshan Hospital of Traditional Chinese Medicine.
